# A Comparison Between Calcium and Strontium Transport by the (Ca^2+^ + Mg^2+^)ATPase of the Basolateral Plasma Membrane of Renal Proximal Convoluted Tubules

**DOI:** 10.3390/membranes15040122

**Published:** 2025-04-12

**Authors:** José Roberto Meyer-Fernandes, Mauro Sola-Penna, Adalberto Vieyra

**Affiliations:** 1 Instituto de Bioquímica Médica Leopoldo de Meis, Centro de Ciências da Saúde, Universidade Federal do Rio de Janeiro, Rio de Janeiro 21941-902, Brazil; 2 Programa de Biologia Estrutural e Bioimagem, Instituto de Bioquímica Médica Leopoldo de Meis, Centro de Ciências da Saúde, Universidade Federal do Rio de Janeiro, Rio de Janeiro 21941-902, Brazil; 3 Laboratório de Enzimologia e Controle do Metabolismo, Faculdade de Farmácia, Centro de Ciências da Saúde, Universidade Federal do Rio de Janeiro, Rio de Janeiro 21941-902, Brazil; maurosp@ufrj.br; 4 Instituto de Biofísica Carlos Chagas Filho, Centro de Ciências da Saúde, Universidade Federal do Rio de Janeiro, Rio de Janeiro 21941-902, Brazil; 5 Centro Nacional de Biologia Estrutural e Bioimagem/CENABIO, Centro de Ciências da Saúde, Universidade Federal do Rio de Janeiro, Rio de Janeiro 21941-902, Brazil; 6 Programa de Biomedicina Translacional/BIOTRANS, UNIGRANRIO, INMETRO, and UERJ-ZO, Duque de Caxias 25071-202, Brazil

**Keywords:** renal proximal tubules, plasma membrane (Ca^2+^ + Mg^2+^)ATPase, Sr^2+^ transport, Ca^2+^ transport

## Abstract

In this work, the utilization of calcium and strontium by the (Ca^2+^ + Mg^2+^)ATPase of the basolateral plasma membrane of renal proximal convoluted tubules were compared. [^90^Sr]Sr^2+^ and [^45^Ca]Ca^2+^ uptake by vesicles derived from this membrane were strictly dependent on ATP and Mg^2+^, and no other nucleotide was able to support the transport. Each cation inhibited the uptake of the other one in a purely competitive fashion (the same Vmax; increased K_0.5_), without causing a significant change in the influx rate. These results indicate that both cations bind at the same transport site on the enzyme, facing the cytosolic surface of the cell. The K_0.5_ for Sr^2+^ obtained for (Sr^2+^ + Mg^2+^)ATPase activity was 13.1 ± 0.2 µM and for Sr^2+^ uptake was 13.4 ± 0.1 µM. They were higher than K_0.5_ for Ca^2+^ obtained for (Ca^2+^ + Mg^2+^)ATPase activity (0.42 ± 0.03 µM) and for Ca^2+^ uptake (0.28 ± 0.02 µM). It is postulated that the lower ATPase affinity for Sr^2+^ is associated with greater steric difficulties for the occupation by this cation of the binding and transport sites, as a consequence of its greater crystal ionic radius (1.13 Å for Sr^2+^ against 0.99 Å for Ca^2+^).

## 1. Introduction

Calcium transport across the basolateral membranes of proximal tubule cells is mediated, in part, by an ATPase that is stimulated by micromolar Ca^2+^ concentrations in the presence of Mg^2+^ [1,2,3]. The renal (Ca^2+^ + Mg^2+^)ATPase is located in the plasma membrane of basolatera1 membranes of proximal tubule cells, where it plays a central role in the regulation of intracellular Ca^2+^ levels. Proximal tubules are responsible for the reabsorption of most of the ultrafiltered Ca^2+^ in the nephron [4]. Thus, these cells must handle a continuous flux of Ca^2+^ and, at the same time, maintain a very low cytoplasmic Ca^2+^ concentration.

Evidence has been presented [3,5,6] showing that the renal (Ca^2+^ + Mg^2+^)ATPase belongs to the P-ATPase class [7], passing through two principal conformational states (E1 and E2) during its catalytic cycle. 

Membrane vesicles from the sarcoplasmic reticulum of skeletal muscle retain a membrane-bound (Ca^2+^ + Mg^2+^)ATPase (SERCA) which is also able to catalyze ATP hydrolysis, stimulated by Sr^2+^ ions, and to transport Sr^2+^ at the expense of ATP hydrolysis [8]. A plasma membrane (Ca^2+^ + Mg^2+^)ATPase (PMCA) with these kinetic characteristics, also able to hydrolyze ATP stimulated by Sr^2+^ and transporting Sr^2+^, has not yet been characterized. Here, for the first time, we are characterizing the Sr^2+^ ion transport and the (Sr^2+^ + Mg^2+^)ATPase activity in the plasma membrane of a cell.

## 2. Materials and Methods

### 2.1. Materials

All the reagents of analytical grade were purchased from Sigma Chemical Co. (St. Louis, MO, USA), or Merck (São Paulo, Brazil). The distilled water used to prepare all the solutions was deionized using a Milli-Q system of resins (Millipore Corp., Burlington, MA, USA). Radioactive Pi (^32^Pi) was purchased from Instituto de Pesquisas Energéticas e Nucleares (São Paulo, Brazil) as orthophosphoric acid and was purified by extraction in a phosphomolybdate complex with a mixture of 2-butanol/benzene followed by re-extraction into the aqueous phase with ammonium hydroxide and then precipitation as a MgNH_4_PO_4_ complex. [**γ**-^32^P]ATP (specific activity 10^4^ Bq/nmol ATP) was prepared as previously described by Glynn and Chappell [9]. Ionized Ca^2+^ and Sr^2+^ concentrations were calculated using a computer program based on an iterative method that takes into account the different species involved in the equilibrium between EGTA, Ca^2+^, Sr^2+^, ATP^4−^, Mg^2+^, H^+^, and Pi [10,11].

### 2.2. Preparation of Purified Vesicles Derived from Basolateral Membranes

Basolateral membrane vesicles were isolated from sheep kidney proximal tubules by the Percoll gradient method [12]. Compared with the initial homogenate, this plasma membrane fraction was enriched 9–12-fold in the specific activity of the basolateral membrane marker (Na^+^ + K^+^)ATPase. Protein concentrations were determined using Folin’s phenol reagent [13] and bovine serum albumin as a standard. 

### 2.3. ^45^Ca^2+^ and ^90^Sr^2+^ Uptake

Except when otherwise noted, the basic medium contained, in a final volume of 0.5 mL, 30 mM Tris-HCl buffer (pH 8.5), 5 mM ATP, 5 mM MgCl_2_, 1 mM ouabain, 10 mM NaN_3_, 0.1 mM EGTA, and 0.1 mM ^45^CaCl_2_ (10 µM free Ca^2+^; specific activity 5.0 × 10^3^ Bq/nmol CaCl_2_) or 0.1 mM ^90^SrCl_2_ (10 µM free Sr^2+^; specific activity 5.0 × 10^3^ Bq/nmol SrCl_2_). The experiments were carried out at 37 °C. ^45^Ca^2+^ or ^90^Sr^2+^ uptake was started by the addition of membranes (protein concentration 0.2 mg/mL) and stopped by Millipore filtration [14], using 0.45 µm pore size filters. The ^45^Ca^2+^ and ^90^Sr^2+^ remaining in the vesicles were counted in a liquid scintillation counter after the filters were washed twice with 10 mL of a cold solution containing 2 mM La(NO_3_)_3_, 100 mM KC1, and 20 mM MOPS-Tris (pH 7.0). ^90^SrCl_2_ and ^45^CaCl_2_ were obtained from New England Nuclear Corporation (Boston, MA, USA). 

### 2.4. (Ca^2+^ + Mg^2+^)ATPase and (Sr^2+^ + Mg^2+^)ATPase Activities

The activities were measured under the same conditions as the ^45^Ca^2+^ and ^90^Sr^2+^ uptakes, except that the reactions were performed in the presence of 5 mM [γ-^32^P]ATP. The reaction was initiated by the addition of membrane and stopped by the addition of 1.0 mL of ice-cold 25% charcoal in 0.1 M HCl to adsorb the non-hydrolyzed [**γ**-^32^P]ATP [15]. Following centrifugation at 4000× *g* for 30 min, an aliquot of the supernatant was withdrawn to measure the amount of ^32^Pi released. Spontaneous hydrolysis of [**γ**-^32^P]ATP was measured in tubes run in parallel in which the enzyme was added after the ice-cold charcoal suspension. The (Sr^2+^ + Mg^2+^)ATPase and (Ca^2+^ + Mg^2+^)ATPase activities were quantified as the difference between the ATP hydrolysis measured in the presence of SrCl_2_ or CaCl_2_, and in its absence (EGTA 1 mM). ^45^Ca, ^90^Sr, and ^32^Pi were counted in a liquid scintillation counter. 

### 2.5. Data Analysis

Data analysis was performed using the software Enzfitter (Elsevier-Biosoft, Cambridge, United Kingdom). Values for the variables were calculated by non-linear regression. Data points represent means ± SEM of three determinations using different membrane preparations. When indicated, the data were analyzed using Student’s *t*-test, using Prism computer software (GraphPad Software Inc., San Diego, CA, USA). The statistically significant difference was set at *p* < 0.05.

## 3. Results and Discussion

### 3.1. Ca^2+^ and Sr^2+^Transport

**Figure 1:**
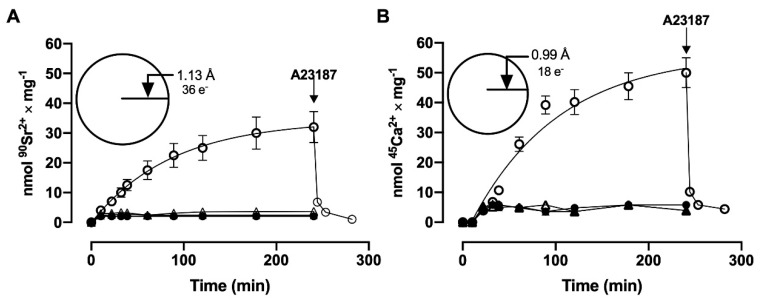
Time course of ^90^Sr^2+^ (**A**) and ^45^Ca^2+^ (**B**) by basolateral plasma membrane vesicles. The reaction medium contained ATP (ο), and sufficient [^90^Sr]SrCl_2_ or [^45^Ca]CaCl_2_ to give a final concentration of 10 μM free Sr^2+^ or 10 μM free Ca^2+^. Modifications of the basic medium were the absence of ATP (●), absence of MgCl_2_ (▲), and replacement of ATP with the non-hydrolyzable analog AMP-P-C-P (Δ). The reaction mixtures were incubated at 37 °C. Arrows indicate the addition of 10 μg/mL of the ionophore A23187. The insets show cartoons of the Sr^2+^ (A) and Ca^2+^ (B) with their respective crystal radii in Å [16] and the corresponding number of electrons.

In order to determine whether Ca^2+^ and Sr^2+^ are actively accumulated in these plasma membrane vesicles, the samples were incubated with ^90^SrCl_2_ or ^45^CaCl_2_ in the presence and absence of ATP, in the presence of a non-hydrolyzable analog of ATP, (5′-adenylyl methylene diphosphonate, AMP-P-C-P), and in the presence and absence of MgCl_2_. Figure 1 shows the time course of ^90^Sr^2+^ (A) and ^45^Ca^2+^ (B) uptake. The transport rates of ^90^Sr^2+^ and ^45^Ca^2+^ by the basolateral membrane vesicles were very slow when compared to those of the sarcoplasmic reticulum [8]. The maximum accumulation level was reached after four hours, which probably reflects the small number of transport units present in the plasma membrane in most cells. At all times, Sr^2+^ uptake was 30–35% lower than Ca^2+^ uptake, and the difference was probably due to a slower rate of active Sr^2+^ influx as a consequence of steric difficulties for the occupancy of the binding and transport sites, because of its greater crystal ionic radius (1.13 Å for Sr^2+^ against 0.99 Å for Ca^2+^) [16]. In these experiments, in the absence of ATP, about 2.1 nmol ^90^Sr^2+^ × mg protein^−1^ and 3.6 nmol ^45^Ca^2+^ × mg protein^−1^ were observed. The same low amounts of accumulated ^45^Ca or ^90^Sr were obtained in the presence of AMP-P-C-P or in the absence of MgCl_2_. It can also be seen in this figure that the addition of A23187, a Ca^2+^ ionophore, induces a rapid and total release of ^90^Sr^2+^ and ^45^Ca^2+^ accumulated by the vesicles, which indicates that ^90^Sr^2+^ as well as ^45^Ca^2+^ are found in the luminal compartment. 

One of the characteristics of plasma membrane (Ca^2+^ + Mg^2+^)ATPases is the high specificity for the energy-donor substrate used for transport, in this case, ATP [17,18]. As shown in Table 1, both ^45^Ca^2+^ transport and ^90^Sr^2+^ transport present the same pattern of selectivity for the different substrates tested.

**Figure 2:**
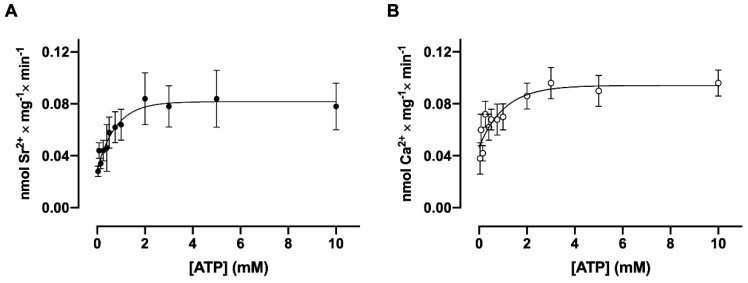
The dependence of ^90^Sr^2+^ (**A**) and ^45^Ca^2+^ (**B**) uptake by basolateral plasma membrane vesicles on ATP concentration. The reaction medium contained the ATP concentrations indicated on the abscissae, and [^90^Sr] SrCl_2_ or [^45^Ca] CaCl_2_ sufficient for a final concentration of 10 μM free Sr^2+^ or 10 μM free Ca^2+^. The reaction mixtures were incubated for 30 min at 37 °C and the rate is expressed in nmol of Sr^2+^ or Ca^2+^ accumulated per mg per min. The mean Km values were calculated from three curves obtained with different preparations. The differences between the two Km values were assessed using Student’s *t*-test.

The dependence of Sr^2+^ and Ca^2+^ uptake on ATP concentration (Figure 2, panel A and panel B, respectively) presents the same stimulatory profile for both ^90^Sr^2+^and ^45^Ca^2+^ uptake with a Km for ATP in the Sr^2+^ transport curve of 0.65 ± 0.06 mM and a Km for ATP in the Ca^2+^ transport curve of 0.52 ± 0.06 mM, without significant differences between these values (*p* > 0.05; Student´s *t*-test). 

### 3.2. Dependence of Sr^2+^ and Ca^2+^ Uptake on Orthophosphate Concentration

**Figure 3:**
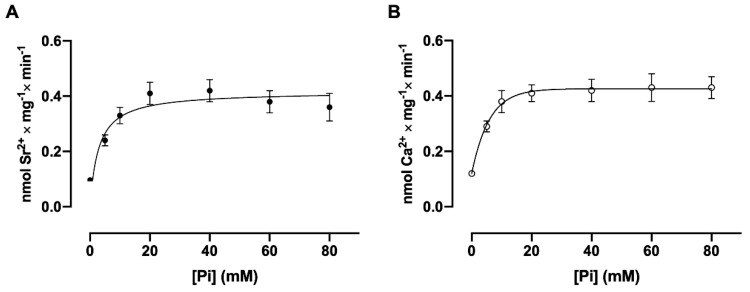
The dependence of ^90^Sr^2+^ (**A**) and ^45^Ca^2+^ uptake (**B**) by basolateral plasma membrane vesicles on Pi concentration. The reaction medium contained the Pi concentrations indicated on the abscissae, and [^90^Sr]SrCl_2_ (A) or [^45^Ca]CaCl_2_ (B) sufficient for a final concentration of 10 μM free Sr^2+^ or 10 μM free Ca^2+^. The reaction mixtures were incubated for 30 min at 37 °C and the rate is expressed in nmol of Sr^2+^ or Ca^2+^ accumulated per mg per min. The mean K_0.5_ values were calculated from three curves obtained with different preparations. Differences between the two Km values were assessed using Student’s *t*-test.

As seen in Figure 3, panels A and B, the dependence on Pi concentration presents the same stimulatory profile for both ^90^Sr^2+^ and ^45^Ca^2+^ uptake. Due to the small volume of the basolateral membrane inside-out vesicles, the accumulated Ca^2+^ and Sr^2+^ can reach sufficiently high concentrations in the lumen of the vesicles to saturate the low-affinity site facing the internal surface of the vesicle, leading to inhibition of the hydrolytic activity of the (Ca^2+^ + Mg^2+^)ATPase [3]. There are several observations showing that the addition of anions such as phosphate (Pi) and oxalate increases the amount of Ca^2+^ accumulated inside plasma membrane and inside sarcoplasmic reticulum membrane vesicles [19]. This increase in the amount of accumulated Ca^2+^ is attributed to the fact that the intravesicular anions form insoluble complexes with Ca, thus reducing the concentration of free Ca^2+^ in the lumen and thus decreasing the occupancy of the low-affinity inhibitory site. As seen in Figure 3, panels A and B, the dependence on Pi concentration presents the same stimulatory profile for both Sr^2+^ and Ca^2+^ uptake with the same K_0.5_ (5.3 mM and 5.1 mM, respectively; Student’s t-test), reaching saturation at a concentration of 40 mM. 

Another possibility that has been suggested is that incubation of the vesicles in media containing potassium phosphate in the pH range 7.0–7.4 would lead to an asymmetric distribution of the anionic species of the buffer due to differences in permeability of H_2_PO_4_^−^ and HPO_4_^2−^ with the generation of a ΔpH with increased Ca^2+^ uptake [3], due to an increase in the intravesicular concentration of H^+^ available for exchange for external Ca^2+^. The existence of Ca^2+^/nH^+^ cotransport was shown in the (Ca^2+^ + Mg^2+^)ATPase of red blood cells [17]. 

### 3.3. Dependence of Sr^2+^ and Ca^2+^ Uptake on Free Sr^2+^ and Ca^2+^ Concentrations, Respectively

**Figure 4:**
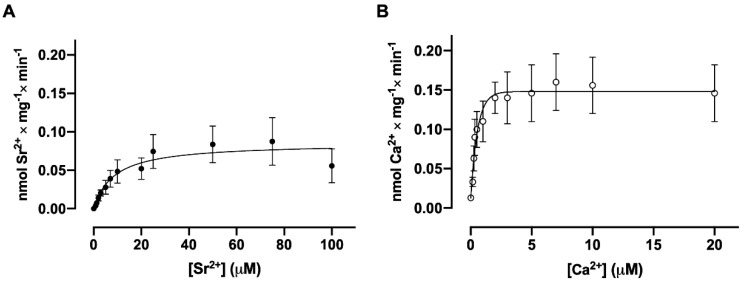
The dependence of ^90^Sr^2+^ and ^45^Ca^2+^ uptake by basolateral plasma membrane vesicles on Sr^2+^ or Ca^2+^ concentrations. The reaction medium contained the concentrations of free Sr^2+^ (**A**) or free Ca^2+^ (**B**) indicated on the corresponding abscissa. The reaction mixtures were incubated for 30 min at 37 °C. The rates are expressed in nmol of Sr^2+^ or Ca^2+^ accumulated per mg per min. The mean K_0.5_ values were calculated from three curves obtained with different preparations. Differences between the two K_0.5_ values were assessed using Student’s *t*-test.

The mechanism of transepithelial Ca^2+^ transport by renal tubular plasma membrane (Ca^2+^ + Mg^2+^)ATPases presupposes that the system located in the membrane is stimulated by micromolar concentrations of the ion, since cytosolic Ca^2+^ concentrations vary in this range. The dependence of Sr^2+^ uptake on Sr^2+^ concentrations follows Michaelis–Menten kinetics, reaching a maximum velocity of 0.11 nmol ± 0.02 nmol Sr^2+^ × mg^−1^ × min^−1^ with a K_0.5_ for Sr^2+^ of 13.4 ± 2.4 µM (Figure 4A). As can be seen in Figure 4B, the dependence of Ca^2+^ uptake on Ca^2+^ concentrations follows the same kinetics, reaching a maximum velocity of 0.15 nmol ± 0.04 nmol Ca^2+^ × mg^−1^ × min^−1^ with a K_0.5_ for Ca^2+^ of 0.28 ± 0.03 µM (Figure 4B). These data show that although the Ca^2+^ active transporter system present in the basolateral plasma membrane can also transport Sr^2+^ with the same velocity, its affinity is greater for Ca^2+^, a cation that has a smaller crystalline ionic radius than Sr^2+^ [16], and which would therefore be able to more easily accommodate the binding located in transmembrane domains of the ATPase molecule [20].

### 3.4. Sr^2+^ and Ca^2+^ Transport Competition Assays

**Figure 5:**
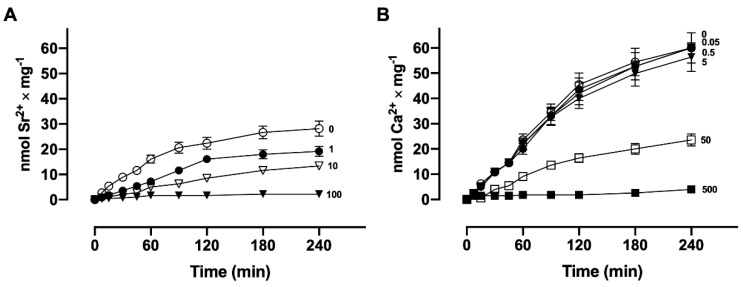
The effects of unlabeled Ca addition on ^90^Sr uptake and of unlabeled Sr addition on ^40^Ca uptake. (**A**): Time course of ^90^Sr accumulation ([Sr^2+^] = 10 μM) in the absence of CaCl_2_ (O), or in the presence of 1 μM (●), 10 μM (▽), or 100 μM (▼) CaCl_2_. (**B**): Time course of ^45^Ca accumulation ([Ca^2+^] = 0.5 µM) in the absence of SrCl_2_ (O), or in the presence of 0.05 μM (●), 0.5 μM (▽), 5 μM (▼), 50 μM (□), or 500 μM (■) SrCl_2_. The reaction mixtures were incubated during the times indicated on the abscissae at 37 ^°^C to measure the corresponding accumulation levels.

If the Ca^2+^ transport system present in the basolateral membrane were the same as that responsible for Sr^2+^ transport, the addition of increasing concentrations of non-radioactive Ca^2+^ or Sr^2+^ should decrease the amount of transported ^90^Sr^2+^ or ^45^Ca^2+^, respectively. In Figure 5A, it is observed that ^90^Sr^2+^ uptake decreases as increasing concentrations of free Ca^2+^ are added simultaneously. The same inhibitory effect can also be observed on ^45^Ca^2+^ uptake when increasing concentrations of free Sr^2+^ are added to the reaction medium (Figure 5B). However, the inhibition promoted by the addition of Sr^2+^ only becomes more evident when a concentration of Sr^2+^ 100 times greater than that of Ca^2+^ is added to the medium. These results are consistent with those showing that the transporter system present in the plasma membrane has greater affinity for Ca^2+^ than for Sr^2+^.

### 3.5. Inhibition of Sr^2+^ Transport by Vanadate 

**Figure 6:**
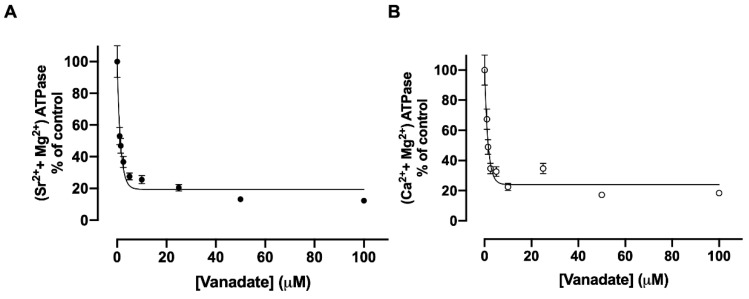
The effect of vanadate on (Sr^2+^ + Mg^2+^)ATPase (**A**) or (Ca^2+^+Mg^2+^)ATPase (**B**) activities from basolateral membranes. The reaction medium contained 10 µM free Sr^2+^ or 10 µM free Ca^2+^ and the vanadate concentrations indicated on the *abscissae*. The reaction mixtures were incubated for 30 min at 37 °C.

**Figure 7:**
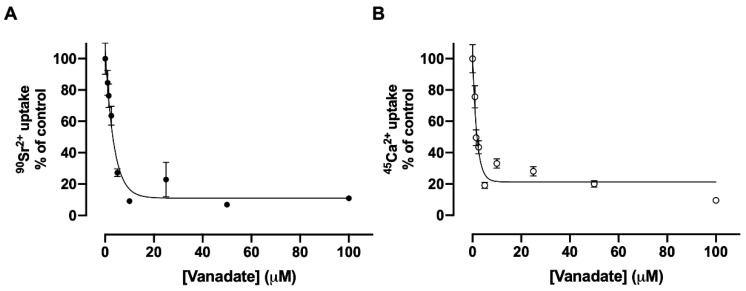
The effect of vanadate on the uptake of ^90^Sr (**A**) or ^45^Ca (**B**) by basolateral plasma membrane vesicles. The reaction medium contained 10 µM free Sr^2+^ or 10 µM free Ca^2+^ and the vanadate concentrations indicated on the *abscissae*. The reaction mixtures were incubated for 30 min at 37 °C.

It has been demonstrated that orthovanadate is capable of inhibiting a large number of transporting ATPases that form a phosphorylated intermediate during their catalytic cycle, defined as P-ATPases [7]. Vanadate is one of the most studied P-type ATPase inhibitors [21]. It is known that the orthovanadate ion VO_3_^-^ presents a great structural similarity to PO_4_^2-^. Figure 6 shows the effects of vanadate on (Sr^2+^ + Mg^2+^)ATPase and (Ca^2+^ + Mg^2+^)ATPase activities of the renal basolateral membrane. Using plasma membranes, the (Sr^2+^ + Mg^2+^)ATPase and (Ca^2+^ + Mg^2+^)ATPase activities are similarly very sensitive to vanadate below 10 μM of the compound (Figure 6, panels A and B). The same inhibitory profile is observed when the concentration of vanadate in Sr^2+^ and Ca^2+^ uptake assays is increased (Figure 7, panels A and B).

### 3.6. pH Dependence Assays

**Figure 8:**
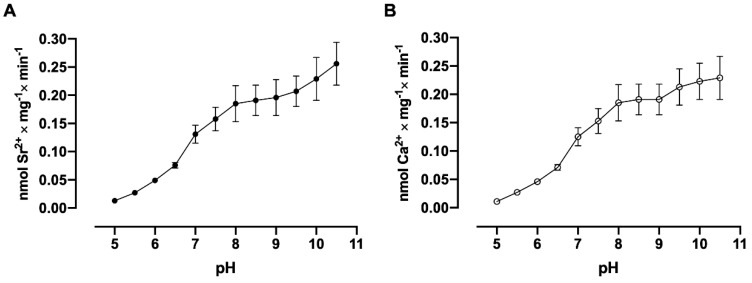
The dependence of ^90^Sr^2+^ (**A**) and ^45^Ca^2+^uptake (**B**) by basolateral plasma membrane vesicles on medium pH. The reaction medium was adjusted to the pH values shown on the *abscissae* by adding HCl or Tris base and contained 10 µM free Sr^2+^ or 10 µM free Ca^2+^. The reaction mixtures were incubated for 30 min at 37 °C.

**Figure 9:**
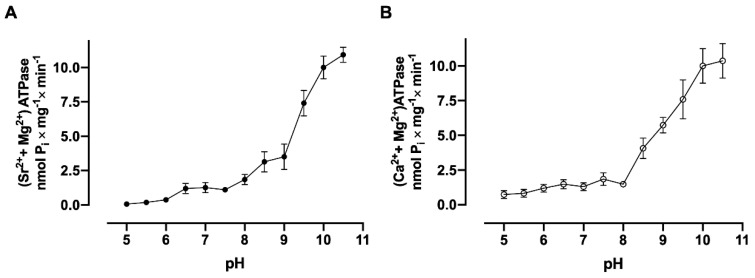
The dependence of (Sr^2+^ + Mg^2+^)ATPase (**A**) and (Ca^2+^ + Mg^2+^)ATPase (**B**) activities from basolateral plasma membranes on medium pH. The reaction medium was adjusted to the pH values shown on the *abscissae* by adding HCl or Tris base and contained 10 µM free Sr^2+^ or 10 µM free Ca^2+^. The reaction mixtures were incubated for 30 min at 37 °C.

Several amino acids in different domains of the (Ca^2+^ + Mg^2+^)ATPase molecule are potential candidates to offer -COOH and -OH groups in their side chains which are capable of contributing to the binding of the Ca^2+^ or Sr^2+^ ions after deprotonation [22]. With the use of renal plasma membrane vesicles, low levels of Sr^2+^ and Ca^2+^ accumulation (Figure 8, panels A and B) were observed at pH 5.0. However, a progressive increase in the velocity of accumulation of both cations was observed due to the increase in pH of the medium. In Figure 9, it is observed that (Sr^2+^ + Mg^2+^)ATPase (Figure 9A) and (Ca^2+^ + Mg^2+^)ATPase (Figure 9B) activities increase as the pH of the medium increases, with a profile similar to that shown for Sr^2+^ and Ca^2+^ uptake in Figure 8, panels A and B.

### 3.7. Specificity for Different Energy-Donor Substrates

It has been described that one of the differences between plasma membrane (Ca^2+^ + Mg^2+^)ATPase (PMCA) and sarcoplasmic reticulum (Ca^2+^ + Mg^2+^)ATPase (SERCA) is the high specificity for ATP [6]. Nucleotides other than ATP are inefficient to sustain Ca^2+^ uptake catalyzed by different plasma membrane (Ca^2+^ + Mg^2+^)ATPases. The high specificity for ATP as an energy donor for Sr^2+^ and Ca^2+^ transport (Table 1), confirms the plasma membrane origin of the vesicles used in this study, and rules out the possibility that Sr^2+^ uptake was due to endoplasmic reticulum membrane vesicles present in the preparation used.

The reaction medium contained 10 μM free Ca^2+^ or 10 μM free Sr^2+^ and 5 mM of the nucleotides indicated in the Table 1. The reaction mixtures were incubated for 30 min at 37 °C.

### 3.8. Conclusions

**Figure 10:**
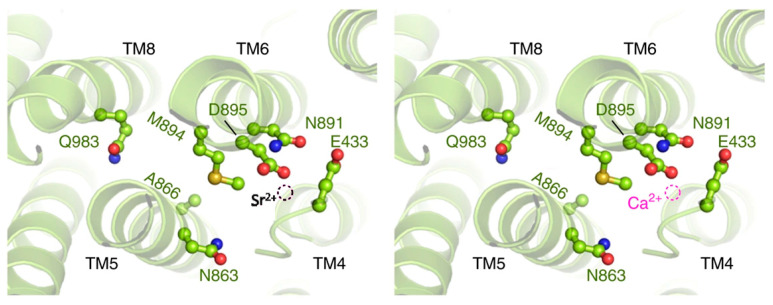
The proposed model for Sr^2+^ binding to the Ca^2+^-binding sites of plasma membrane (Ca^2+^ + Mg^2+^)ATPase. Adapted from Ref. [20]. Left: a representation of Sr^2+^ binding. Right: a representation of Ca^2+^ binding. The coordinating residues D895, N891, E433, and M894 align to allow Sr^2+^ binding and access to the transmembrane channel. The figure also depicts the transmembrane domains TM4, TM5, and TM6. The view is from the cytoplasmic side. The larger radius of Sr^2+^ [16] perturbs its proper alignment and, consequently, the kinetic properties of its transport. Modified from Gong et al. [20] and reproduced under the terms of the Creative Common License CC BY 4.0.

From the results described and discussed in Figure 1, Figure 2, Figure 3, Figure 4, Figure 5, Figure 6, Figure 7, Figure 8 and Figure 9, we propose (Figure 10) that the electronic configuration of Sr^2+^ perturbs its proper alignment with the aspartic acid (D895), asparagine (N891), glutamic acid (E433), and methionine (M894) residues of the (Ca^2+^ + Mg^2+^)ATPase plasma membrane molecule and, consequently, the kinetic properties of its transit through the channel formed by the transmembrane TM4, TM5, and TM6 domains [20]. These results can help explain how the electronic configuration underlies modifications of cation flux properties across biological membranes. Mutation of the residues above to investigate whether Sr^2+^ transport still occurs constitutes a future direction for this study.

## Figures and Tables

**Table 1:** Specificity for different energy-donor substrates in ^90^Sr^2+^ and ^45^Ca^2+^ uptake assays.

Substrates	Uptake of ^90^Sr (%)	Uptake of ^45^Ca (%)
ATP	100	100
CTP	2.4	5.2
GTP	2.7	1.4
ITP	1.6	4.5
UTP	1.4	2.7

Abbreviations: ATP: Adenosine triphosphate; CTP: Cytidine triphosphate; GTP: Guanosine triphosphate; ITP: Inosine triphosphate; UTP: Uridine triphosphate.

## Data Availability

The datasets generated and/or analyzed during the current study are available from the corresponding authors on reasonable request.
